# Inhibitory effects of fenretinide metabolites N-[4-methoxyphenyl]retinamide (MPR) and 4-oxo-N-(4-hydroxyphenyl)retinamide (3-keto-HPR) on fenretinide molecular targets β-carotene oxygenase 1, stearoyl-CoA desaturase 1 and dihydroceramide Δ4-desaturase 1

**DOI:** 10.1371/journal.pone.0176487

**Published:** 2017-04-27

**Authors:** Eugenia Poliakov, William Samuel, Todd Duncan, Danielle B. Gutierrez, Nathan L. Mata, T. Michael Redmond

**Affiliations:** 1 Laboratory of Retinal Cell and Molecular Biology, National Eye Institute, National Institutes of Health, Bethesda, Maryland, United States of America; 2 Acucela Inc., Seattle, Washington, United States of America; University of Florida, UNITED STATES

## Abstract

The therapeutic capacity of fenretinide (N-[4-hydroxyphenyl] retinamide; 4-HPR) has been demonstrated for several conditions, including cancer, obesity, diabetes, and ocular disease. Yet, the mechanisms of action for its pleiotropic effects are still undefined. We hypothesized that investigation of two of the major physiological metabolites of fenretinide, N-[4-methoxyphenyl]retinamide (MPR) and 4-oxo-N-(4-hydroxyphenyl)retinamide (3-keto-HPR), might begin to resolve the multifaceted effects of this synthetic retinoid. We analyzed the effects of fenretinide, MPR, 3-keto-HPR, and the non-retinoid RBP4 ligand A1120, on the activity of known targets of fenretinide, stearoyl-CoA desaturase 1 (SCD1) and dihydroceramide Δ4-desaturase 1 (DES1) in ARPE-19 cells, and purified recombinant mouse beta-carotene oxygenase 1 (BCO1) in vitro. Lipids and retinoids were extracted and quantified by liquid chromatography-mass spectrometry and reversed phase HPLC, respectively. The data demonstrate that while fenretinide is an inhibitor of the activities of these three enzymes, that 3-keto-HPR is a more potent inhibitor of all three enzymes, potentially mediating most of the in vivo beneficial effects of fenretinide. However, while MPR does not affect SCD1 and DES1 activity, it is a potent specific inhibitor of BCO1. We conclude that a deeper understanding of the mechanisms of action of fenretinide and its metabolites provides new avenues for therapeutic specificity. For example, administration of 3-keto-HPR instead of fenretinide may be preferential if inhibition of SCD1 or DES1 activity is the goal (cancer), while MPR may be better for BCO1 modulation (carotenoid metabolism). Continued investigation of fenretinide metabolites in the context of fenretinide’s various therapeutic uses will begin to resolve the pleotropic nature of this compound.

## Introduction

Fenretinide (N-[4-hydroxyphenyl]retinamide (4-HPR)) is a synthetic retinoid originally synthesized in the late 1960s. Fenretinide was first described as a novel retinoid for therapy of breast cancer in rat in 1979 [1]. Fenretinide did not accumulate in the liver and therefore caused little hepatic toxicity in animals [1]. Furthermore, fenretinide accumulates mostly in the mammary gland and is metabolized by mammary epithelial cells in both rodents [2,3] and humans [4]. Since then, a favorable toxicity profile compared to other retinoids has led to extensive study of fenretinide in chemoprevention trials [5,6]. However, fenretinide’s efficacy is limited to premenopausal women with stage I breast cancer, reducing the risk of a second tumor [7], but apparently also reducing incidence of ovarian cancer during the 5-year intervention period [8]. The mechanism of fenretinide’s action is still unresolved. The upregulation by fenretinide of the expression of RARβ and RARβ was associated with fenretinide’s antiproliferative action in ovarian cancer cells [9]. However, the ability of fenretinide to induce apoptosis in cells that are resistant to all-*trans*-retinoic acid suggests that this activity may not involve retinoid receptors in some cell types and may be non-genomic in action [10–12]. Unlike the endogenous retinoids, fenretinide binds poorly to RARs, and several alternative mechanisms of apoptosis induction have been proposed, including an increase in reactive oxygen species [13], ceramide [14] or dihydroceramide [15] accumulation, and, notably, transglutaminase induction that sensitizes cells to Ca^2+^-mediated apoptosis [16,17].

Additionally, fenretinide increases insulin sensitivity and improves glucose intolerance in obese rodents [18]. Fenretinide lowers RBP4 (serum retinol–binding protein) levels in rodents and humans by disrupting the ternary complex of retinol-RBP4-transthyretin [19,20], thereby promoting renal clearance of RBP4 [21]. Long-term therapy with fenretinide partially prevents or reverses obesity, insulin resistance, and hepatic steatosis in mice on a high-fat diet. However, the anti-adiposity effect appears to be independent of the RBP4-lowering effect because in RBP4 knockout mice fenretinide also reduced high fat diet-induced increase in adiposity and hyperleptinemia [22]. Recently, it was shown that fenretinide improves insulin sensitivity, at least in part, by blocking ceramide biosynthesis and inhibiting DES1 [23]. Currently, fenretinide is in a phase II trial for treatment of insulin resistance in obese humans with hepatic steatosis [24].

Additionally, fenretinide was found effective in slowing lesion growth in patients with the geographic atrophy form of age-related macular degeneration (AMD) in a phase two clinical trial [25]. The effects of fenretinide on vision and bisretinoid accumulation in the eye are attributed to its ability to reduce plasma levels of RBP4 and retinol. Recently, however, the role of RBP4 in mediating visual fenretinide effects was questioned as fenretinide treatment of both WT and *Rbp4*^*-/-*^ animals showed comparable levels of 11-*cis*-retinal regeneration [26]. On the other hand, all-*trans*-retinal can be synthesized locally in the tissues by the cleavage of β-carotene. Thus, BCO1 might supply all-*trans*-retinal as an accessory source of vitamin A for the visual cycle [27]. We recently demonstrated that fenretinide is a strong non-competitive inhibitor of BCO1 and therefore some of fenretinide’s effects on vision may be mediated by BCO1 [28]. Also, we have found that fenretinide induces degradation of SCD1 in human RPE cells [29].

Considering fenretinide’s pleiotropic effects, most without clear mechanisms, it would be useful to determine if the major physiological metabolites of fenretinide (MPR and 3-keto-HPR) [30–32] exert some or all activities and could be used more specifically than fenretinide. Additional minor metabolites of 4-HPR were recently described in human and mice plasma including dehydrogenated 4-HPR, monohydroxy-3-keto-HPR as well as further glucuronidated or sulfated metabolites [30]. Metabolism of fenretinide is somewhat different in mice and humans: 3-keto-HPR (4-oxo-4-HPR) is a more prevalent product in mice (~23% of plasma levels of 4-HPR after 4 hours of treatment) while MPR is a major product in humans (~45% of plasma levels of 4-HPR) [30]. 3-keto-HPR is a polar fenretinide metabolite that more effectively kills cancer cells than 4-HPR, inhibits fenretinide-resistant cell growth, and acts synergistically in combination with the parent drug [33]. Besides acting as an antimicrotubule agent, 3-keto-HPR induced apoptosis through a signaling cascade starting from ROS generation and involving endoplasmic reticulum (ER) stress response [34]. Here we demonstrate that proteins affected by fenretinide respond differentially to fenretinide metabolites.

## Experimental methods

### Materials

Fenretinide and 3-keto-fenretinide were obtained from TRC, Inc. (Toronto, ON). A monoclonal antibody to SCD was obtained from Kamiya Biomedical Company (Seattle, WA). The enhanced chemiluminescence (ECL) detection system and peroxidase-conjugated anti-rabbit and anti-mouse antibodies were from GE Healthcare Life Sciences (Piscataway, NJ). The fenretinide analogues N-[4-methoxyphenyl]retinamide (MPR) and N-[4-ethoxyphenyl]retinamide (EPR) were synthesized as described by the Robert W. Curley, Jr., group [35]. The nonretinoid compound, A1120 was synthesized according to a previously described method [36].

### BCO1 activity

Recombinant His-tag mouse BCO1 was produced in *E*.*coli* and purified using Talon CellThru (Clontech Labs, Inc., Mountain View, CA) resin as described previously [37]. β-carotene was delivered in 1% octylthioglucoside. Enzyme activities were measured in the presence of fenretinide in DMSO or DMSO alone (not more than 5% of total volume). All other inhibitors were delivered in ethanol.

### A2E mass spectrometry analysis

Samples were prepared in the same manner as previously published [38]. Eyecups were dissected from fresh eyes, removing the retina when possible, and stored at -80°C. For each sample, 4–6 eyecups were combined. Extractions were done in red light, on ice. First, a glass-glass tissue homogenizer was rinsed with 1:1 chloroform:methanol and 1x PBS and then eyecups, in 1 mL of 1x PBS, were added to the homogenizer. The sample tube was rinsed with 0.5 mL of 1x PBS, which was added to the homogenizer. Next, 2 mL of 1:1 chloroform:methanol was added to the homogenizer and the tissue was ground until only tiny pieces of intact tissue remained (around 20 times). Homogenized tissue was poured into a glass vial, and the homogenizer was rinsed with 1 mL of 1:1 chloroform:methanol and 0.5 mL of 1x PBS. The rinse was poured into the glass sample vial. The homogenizer was subsequently rinsed with 1 mL of chloroform and finally with 1 mL of methylene chloride. Rinses were poured into the sample vial. The sample was vortexed for 1 min and then spun for 5 minutes at 13,000 *g*. The bottom organic layer was pipetted to a new glass vial, dried under argon, and stored at -80°C. Immediately prior to MS analysis, the sample was reconstituted in 40 μL of 100% MeOH, 0.1% TFA.

High-performance liquid chromatography mass spectrometry (HPLC-MS) analysis was performed on a Waters Alliance 2695 HPLC in line with an AB Sciex API2000 Triple quadrupole mass spectrometer. Sample aliquots, 15 μL, were injected via a Waters 2695 separation module autosampler (held at 10°C) onto an Imtakt, Cadenza C18 column, 150 x 2.0 mm (length x i.d.), 5 μm particle size, held at 25 ± 5°C. Mobile phase A (MPA) consisted of 100% water, 0.1% formic acid, and mobile phase B (MPB) was 100% methanol, 0.1% formic acid. Analytes were separated at 0.2 mL/min using the following gradient: 0–5 minutes, 15% MPA, 85% MPB; 5–15 minutes, 85–100% MPB; 15–45 minutes, 100% MPB. These chromatography parameters are similar to previously published work [38] but use methanol instead of acetonitrile as the mobile phase. Column washing with methanol and equilibration was performed in between sample runs. A2E, isoA2E, and oxidized A2E were monitored in positive ion mode via multiple reaction monitoring using the following transitions: for A2E/iso-A2E, *m/z* 592.5 → 358.5, *m/z* 592.5 → 402.5, and *m/z* 592.5 → 376.6. All transitions had a declustering potential (DP), focusing potential (FP), and entrance potential (EP) of 91, 120, 12, respectively, and the collision energy for each transition was 70, 61, and 62, respectively. For oxidized A2E, transitions were as follows: *m/z* 608.5 → 444.7, and *m/z* 608.5 → 404.4. All transitions had a DP, FP, and EP of 106, 370, and 10, respectively, and the collision for each transition was 65 and 58, respectively. Data were analyzed using Analyst Software, version 1.5.2. For quantitation, total A2E (the sum peak areas for A2E and isoA2E) was compared to a standard curve of synthesized A2E (0.15–15 pmol) run in triplicate.

### SCD1 analysis

#### Cell culture

Human retinal pigment epithelial cells (ARPE-19) were grown in DMEM/F12 1:1 media with 5% FBS (Life technologies) and 1% Antibiotic/antimycotic solution (Life technologies) treated with 10 μM of either fenretinide, MPR, 3-keto-HPR, or A1120. Compounds were dissolved in dimethyl sulfoxide (DMSO) and then added to the cell culture medium. Cells designated as control received an equal amount of dimethyl sulfoxide. After 16 h, cells were incubated with 50 μM palmitic acid triply deuterated (D3) on the methyl group (Cambridge Isotope Laboratories, Tewksbury, MA) for an additional 5 h before harvesting.

#### Lipid extraction and liquid chromatography mass spectrometry

Cells were detached from culture plates using 0.05% Trypsin-EDTA, washed with PBS, and pelleted. Cell pellets were resuspended in 450 μl Cell Lysis Buffer (Cell Signaling Technology, Danvers, MA) then sonicated on ice until uniformly dispersed. An aliquot (50 μl) of the dispersed cell suspension was used for protein quantitation, and the remaining 400 μl was used for lipid extraction as follows. Samples were spiked with a small volume of pentadecanoic acid (in CHCl_3_) as internal standard, and were then subjected to saponification (by addition of an equal volume of 0.6 M methanolic KOH and incubating at 72°C for 15 minutes). Following saponification, samples were acidified by addition of 100 μl formic acid. The acidified mixture was extracted twice with 1 ml chloroform to recover free fatty acids. The chloroform extracts were pooled, solvent was evaporated under argon at 37°C, and the residue was resolubilized in 100 μl chloroform/methanol (1:4) for analysis by liquid chromatography mass spectrometry (LC/MS) using a modification of the procedures of Dillon et al. [39]. The LC-MS system consisted of a 1200 Series Capillary LC (Agilent, Santa Clara, CA) coupled to a Micromass Q-Tof micro mass spectrometer (Waters Corp., Milford, MA) equipped with an electrospray ionization source. Separation of fatty acids was achieved by isocratic elution on a PLRP-S polymeric reversed-phase column (100 Å, 3 μm, 2.1 x 150 mm; Agilent) heated to 65°C. A mobile phase consisting of 25% acetonitrile:chloroform (1:1), 40% methanol, 25% water, 10% 10 mM ammonium acetate was run at a flow rate of 0.1 ml/min. The mass spectral analysis of the fatty acids was performed in the negative ion mode using the following instrument settings: capillary voltage, 1600; sample cone voltage, 40; cone gas flow, 40 L/hr; desolvation gas flow, 400 L/hr; source temperature, 80°C; desolvation temperature, 100°C. Data was then analyzed using MassLynx software v 4.1 (Waters) by extracting the ion chromatograms of the labeled palmitic and palmitoleic acid peaks and measuring peak area.

#### Western blot analysis

Samples were resuspended in 20 μl 4X reducing NuPAGE LDS sample buffer (Invitrogen). For western blot analysis, equal amounts of total protein (50 μg) from cell extracts were subjected to SDS-polyacrylamide gel electrophoresis using 4–12% NUPAGE Bis-Tris gels and then transferred to a nitrocellulose membrane using iBlot^®^ Dry Blotting System (Invitrogen, Carlsbad, CA). After blocking in 5% non-fat milk in Tris-buffered saline (TBS) containing 0.01% Tween 20 for 1 h, the membranes were incubated overnight at 4°C with mouse monoclonal anti-SCD1 antibody (1:200; Kamiya Biomedical Company). Peroxidase-conjugated goat anti-mouse IgG antibody was used as secondary antibody. Immunocomplexes were visualized by a chemiluminescence method using the ECL Prime western blotting Detection Kit (GE Healthcare Life Sciences). The blots were then stripped and reprobed with mouse monoclonal anti-α-tubulin antibody.

### Ceramide analysis

#### Cell culture and lipid extraction

ARPE-19 cells were seeded onto tissue culture plates at a density of 2x10^5^ cells/ml in complete medium and allowed to grow overnight. Cells were then treated with retinoids (10 μM) or vehicle (control) for 24 hours in a 37°C, 5% CO_2_ incubator at which time cells were harvested and total lipids were extracted into methyl-tert-butyl ether (MTBE) using the method of Matyash et al. [40]. Extracted lipids normalized to total protein as described [41] were subjected to liquid chromatography mass spectrometry (LC/MS) to determine the sphingolipid content in treated ARPE-19 cells. LC/MS analysis was performed using a Waters 2695 LC system coupled to an AB Sciex API2000 triple quadrupole mass spectrometer. Sphingolipids were resolved on a Pursuit Diphenyl reversed-phase column (3 μm, 2.0 x 50 mm; Agilent) using a 3 min linear gradient from 30 to 95% mobile phase B in mobile phase A, beginning 1 min after injection at a flow rate of 0.8 ml/min (mobile phase A: 25 mM ammonium acetate / 0.1% formic acid; mobile phase B: acetonitrile / 0.1% formic acid). The mass spectral analysis of the sphingolipid species was performed using electrospray ionization in the positive ion mode with multiple reaction monitoring (MRM). The following compound-specific transitions for precursor and characteristic product ions were used: 538 → 264 for C16 Ceramide (Cer); 540 → 266 for C16 dihydroceramide (DHCer); 566 →264 for C18 Cer; and 568 → 266 for C18 DHCer. Data was acquired and analyzed using Analyst software v 1.5 (AB Sciex).

### Statistical analysis

To test for significant differences between treatment groups in ceramides and fatty acid analysis, two-way ANOVA was used. Dunnett’s multiple comparison test was used to determine whether there were significant differences between individual treatment groups relative to the controls. The alpha level for significance was P< 0.05. Values presented means ± SD (n = 3)

### Protein quantitation

Protein concentration was determined using the DC Protein Assay Kit (Bio-Rad Laboratories, Hercules, CA).

### Animals

The C57BL/6 mice were obtained from Charles River (USA). *Bco1*^*-/-*^ mice were obtained from Royal DSM Company (Heerlen, Netherlands). All procedures concerning animals were in accordance with institutional regulations and with the statement of the Association for Research in Vision and Ophthalmology for the use of animals in research and were carried out under an institutional Animal Study Protocol approved by the National Eye Institute (NEI), NIH Animal Care and Use Committee.

The mice were housed under controlled conditions at a room temperature of 22–24°C, with 30–75% relative humidity, a 14/10 hr controlled light/dark cycle at a light intensity of less than 30 lux inside the cage. The mice were housed in groups of 3–5 (siblings of same sex) on IVC racks and in cages made by Lab Products, Inc. (individually ventilated). Each cage was provided with about 0.5 inches deep of Envigo 7090M Teklad maple sani chips with one nestlet. Chlorinated water and food-pellets (NIH-31, Harland) were provided ad lib. At the end point of the experiment, mice were euthanized with 100% CO2 gas at a gradual filling rate to minimize the discomfort of the animals.

### RARbeta agonist and antagonist assay

The potency of agonists and antagonists was measured by GeneBLAzer RAR-beta DA Assay Kit (ThermoFisher Scientific) according to the manufacturer’s recommendations.

## Results and discussion

### Fenretinide metabolites inhibit activity of BCO1 enzyme

**Fig 1:**
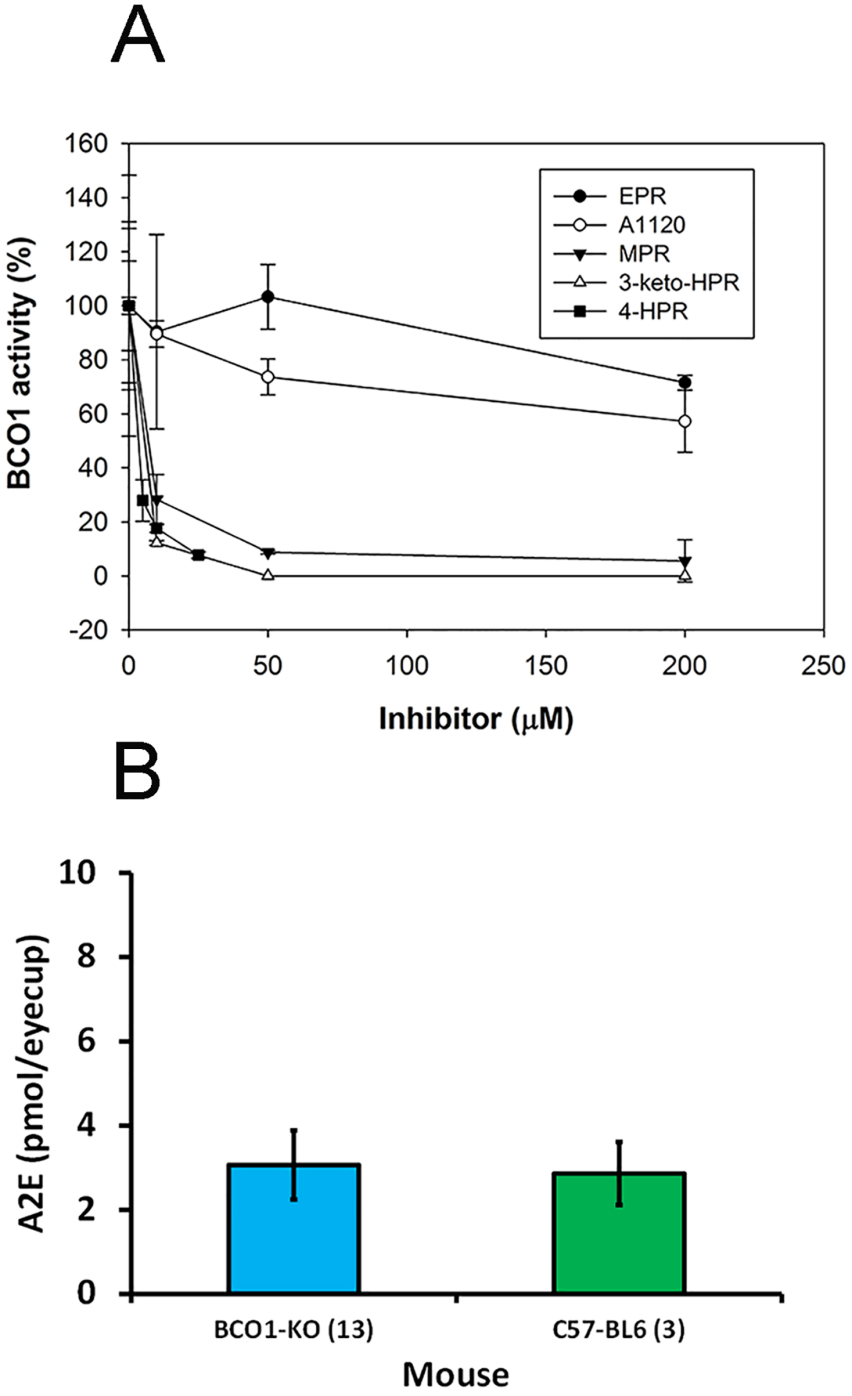
**A) In vitro inhibition of recombinant mouse BCO1 activity by HPR, its metabolites, EPR and A1120**. Inhibitor (0–200 μM) was added from a 10 mM stock in DMSO (4-HPR) or 20 mM stock in ethanol (A1120, EPR, MPR, 3-keto-HPR) to an *in vitro* reaction mixture prior to addition of substrate. The final concentration of ethanol in all samples was 1%. The enzyme was preincubated with the inhibitor for 10 min at 4°C in the standard reaction buffer and was then added to 20 μM β-carotene and activity determined as described in Methods. Data plotted as means±standard deviation; n = 3; **B) Accumulation of total A2E bisretinoid in the eyes of aged (1 year old) mice.** Analysis was done in C57BL/6 wt (3 animals) and *Bco1*^*-/-*^ mice (13 animals).

Previously, we have found fenretinide to inhibit BCO1 activity *in vitro* [28,42]. However, some of the *in vivo* effects of fenretinide may be mediated by its physiological metabolites. To date, two major metabolites have been described in the literature: N-[4-methoxyphenyl]retinamide (MPR) and 4-oxo-N-(4-hydroxyphenyl)retinamide (3-keto-HPR) [30–32]. When tested in vitro on recombinant mouse BCO1 protein, we found that both MPR and 3-keto-HPR are potent inhibitors of BCO1 (IC_50_ = 5 and 4 μM respectively; Fig 1A). N-[4-ethoxyphenyl]retinamide (EPR), a synthetic analog of MPR with the bulkier ethoxy group replacing the methoxy group, does not inhibit BCO1 activity (Fig 1A). The non-retinoid ligand A1120, which specifically blocks the binding of retinol to RBP4 [36], also does not affect BCO1 activity, either (Fig 1A). Although BCO1 could serve as a local source of all-*trans*-retinal in the retina, where we have found BCO1 to be expressed [43], and be a target of fenretinide interference, we find that bisretinoid A2E accumulation in *Bco1*^*-/-*^ mouse is not attenuated compared to WT C57BL6 mice (Fig 1B), indicating that this is not a significant source of all-*trans*-retinal in the retina. However, as total A2E levels seem to be low due to signal suppression by sample matrix, there could be changes in total A2E production that we could not detect.

### SCD1 and DES1 activity are affected by fenretinide and its metabolite 3-keto-HPR

**Fig 2:**
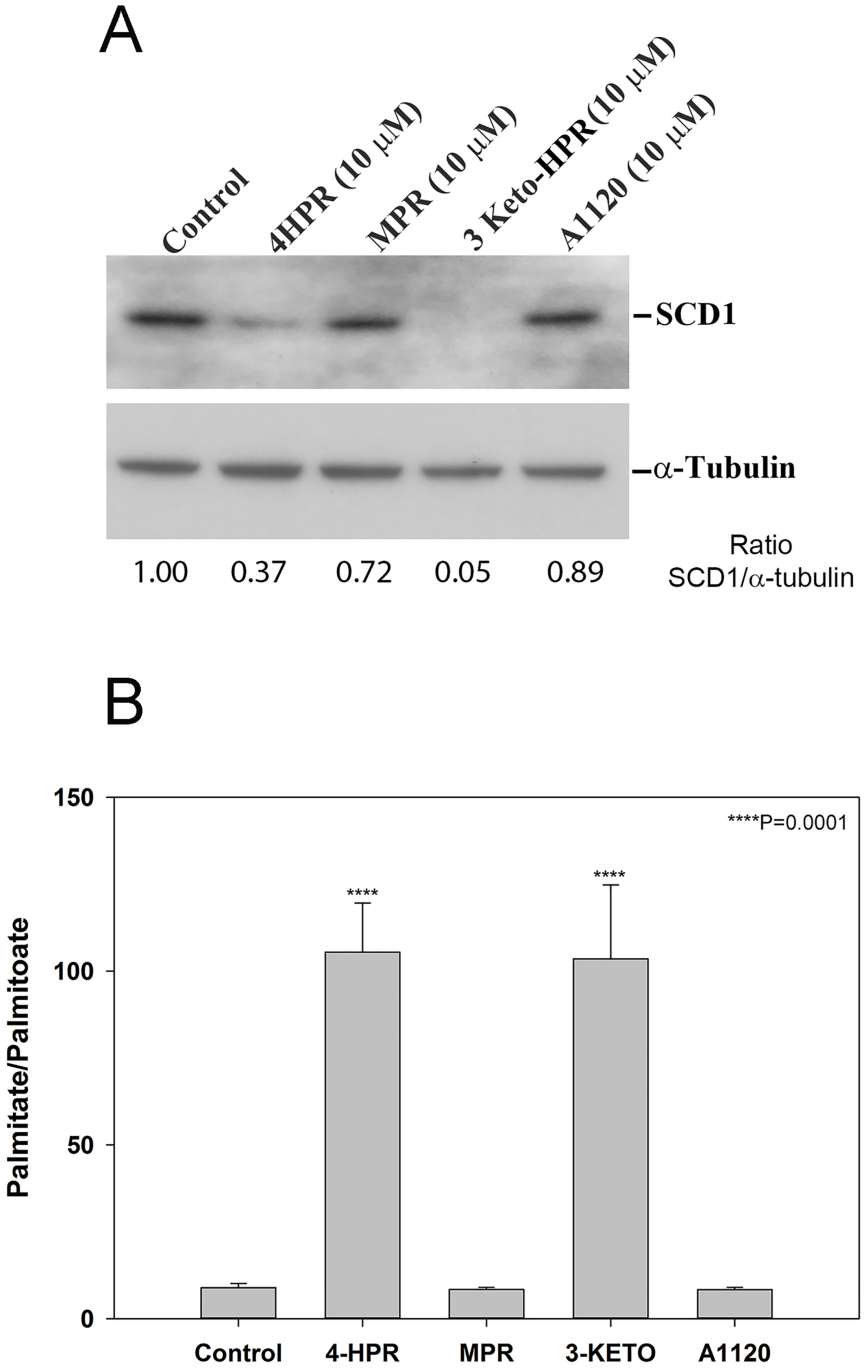
Effect of fenretinide and its metabolites on SCD expression and activity. A) SCD1 protein expression is decreased after treatment with fenretinide, and its metabolite 3-keto-HPR. ARPE-19 cells were treated with the indicated concentration of fenretinide, its metabolites, A1120, or vehicle for 24 h. Cell lysates were analyzed by Western blotting using mouse monoclonal antibody against SCD1 (upper panel) in comparison with immunoreactivity to α-tubulin which was employed as a loading control (lower panel) and densitometry for each lane was performed and analyzed by ImageJ to provide the SCD1/α-tubulin ratio for each lane. B) SCD1 protein activity is decreased after treatment with fenretinide, and its metabolite 3-keto-HPR. ARPE-19 cells were treated with the indicated concentration of fenretinide, its metabolites, A1120, or vehicle for 16 h, then incubated an additional 5 h in the presence of methyl D3-palmitic acid (50 μM) before harvesting. Free fatty acids present in lipid extracts were analyzed by LC/MS and SCD1 activity, measured by the conversion of methyl-D3 palmitic acid to methyl-D3 palmitoleic acid, was determined. Fenretinide and 3-keto-HPR are effective inhibitors of SCD1 activity, whereas MPR and A1120 have no inhibitory effect on SCD1 activity at all. Lipids were extracted from two 100 mm Petri dishes and combined for mass spectrometry analysis. Data plotted as means ± standard deviation; n = 3.

We previously found that a SCD1 specific inhibitor could prevent fenretinide-induced apoptosis of ARPE-19 cells [44]. Later we determined that fenretinide inhibits SCD1 expression through ubiquitin-mediated proteasomal degradation and directly inhibits SCD1 activity in ARPE-19 cells [29]. A similar effect of fenretinide on SCD1 activity was found in an animal model of diabetes [45]. In the present experiments we compared the effect of fenretinide and its metabolites on SCD1 expression and activity in ARPE-19 cells. We confirm that fenretinide has an inhibitory effect on SCD1 expression and activity, and we further elucidate that 3-keto-HPR, its physiological metabolite, more efficiently inhibits SCD1 expression (Fig 2A) and activity (Fig 2B) than fenretinide itself, and thus may be the major mediator of the effects of fenretinide on SCD1. On the other hand, MPR and A1120 do not affect either SCD1 expression or activity.

The inhibitory effect of fenretinide and its metabolite 3-keto-HPR on DES1 activity has already been described in the literature [42]. The ratio of substrate to product for C16 and C18 ceramides (dihydroceramide/ceramide) in the presence of DMSO or inhibitors was determined by LC/MS. We found that presence of MPR or A1120 do not lead to overaccumulation of substrate and increase of substrate/product ratio compared to DMSO control for C16 ceramide, and led only moderate increase of C18 ceramide ratio (~2 fold) in the presence of A1120. Thus, A1120 may moderately affect the substrate specificity of the DES1 enzyme.

**Fig 3:**
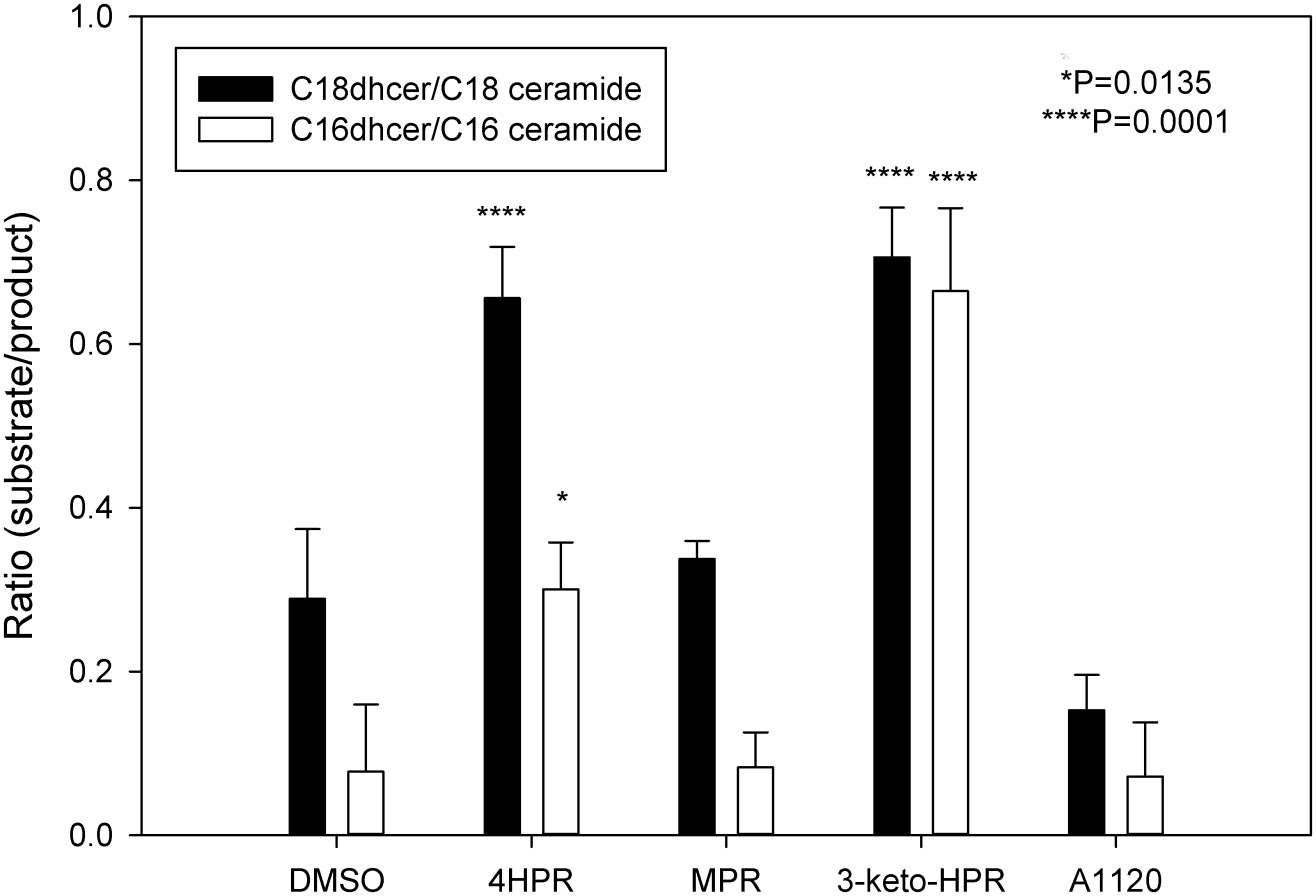
Effect of fenretinide and its metabolites on DES1 activity. ARPE-19 cells were treated with fenretinide, its metabolites, A1120 (all at 10 μM) or vehicle for 24 h, and endogenous C16 and C18 ceramides measured by LC/MS. Suppression of DES1 activity by fenretinide and its metabolites is confirmed by the higher substrate/product (dihydroceramide/ceramide) ratio relative to the DMSO control. The presence of fenretinide or 3-keto-HPR led to a greater accumulation of C16 and C18 dihydroceramide than did MPR or A1120. Lipids were extracted from one 100 mm Petri dish for each biological replicate and subjected to mass spectrometry analysis. Data plotted as means±standard deviation; n = 3.

The presence of fenretinide or 3-keto-HPR led to ~2.7 and ~3 fold increases in ratio for C16 and C18 ceramides ratio respectively. We confirm here that 3-keto-HPR and fenretinide inhibit DES1 activity while MPR and A1120 do not (Fig 3).

The non-retinoid RBP4 inhibitor A1120 did not affect any of the studied enzymes. MPR affinity for RBP4 was found to be 2-fold lower (~100 nM) than for fenretinide or retinol (~30–60 nM) [46]. The kinetics of EPR binding to RBP4 is similar to that of MPR, but it is a less potent inhibitor (data not shown). The binding of 3-keto-HPR to RBP4 has not been studied but it would likely not fit into the RBP4 binding pocket due to potential steric/charge hindrance from the keto moiety.

### Binding of fenretinide metabolites to RARβ

**Fig 4:**
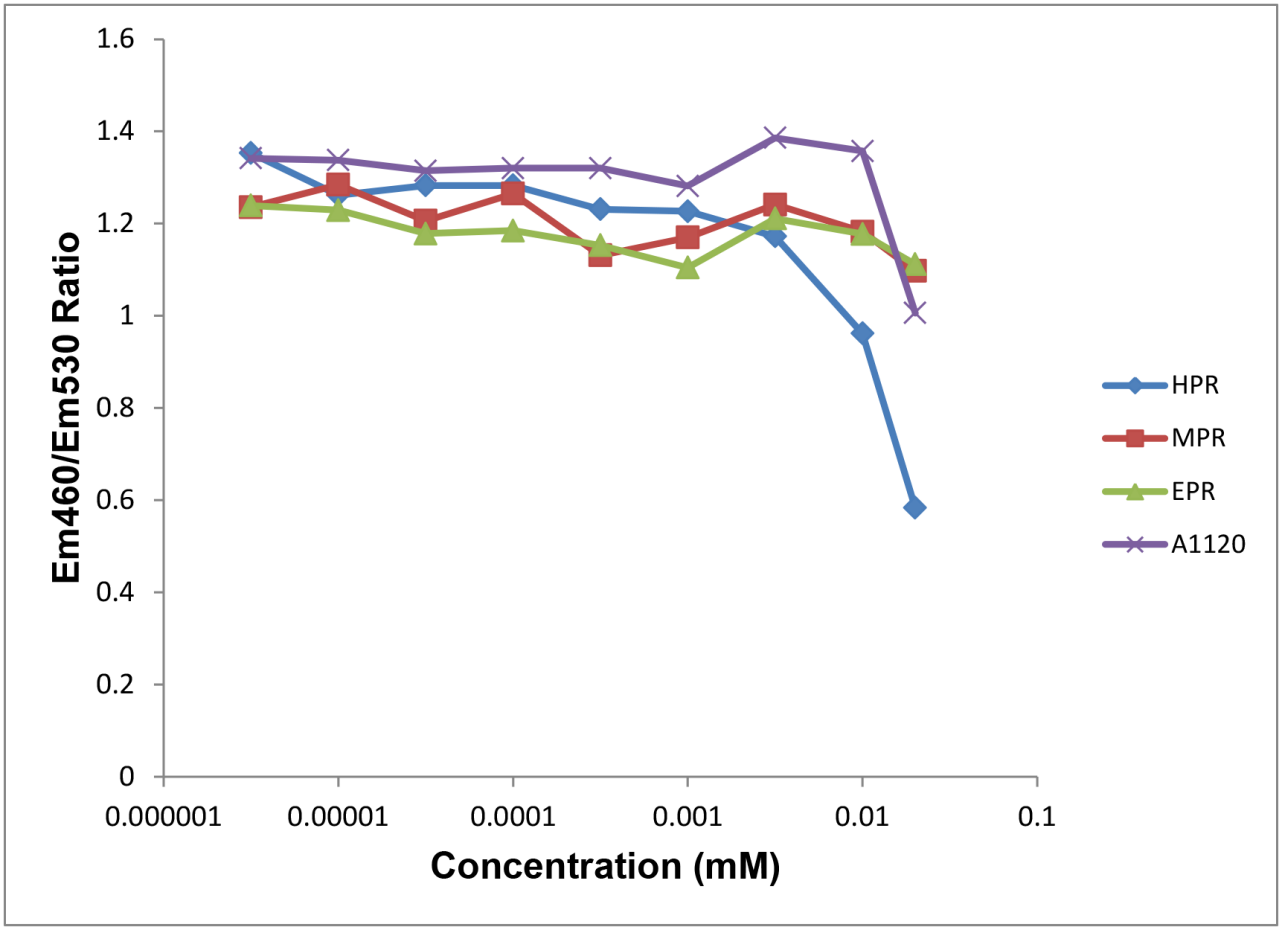
Phenylretinamides and A1120 do not compete or compete poorly with 1 nM at-RA in RARbeta antagonist assay. Fenretinide is the only tested compound that appears to compete, exhibiting an IC_50_ = 10 μM. Fluorescence was measured at excitation (ex) 409 nm/emission (em) 460 and at ex 409nm/em 530 nm and the ratio was determined and plotted against a range of concentrations (10^−5^ to 10^−9^ M) of the tested compounds.

The phenylretinamides may have the ability to modulate the expression of proteins and induce cell apoptosis via RAR-dependent and -independent pathways. Fenretinide effectively induces apoptosis in hepatocellular carcinoma Huh-7 cells by transactivating the RXRα/RARβ-mediated pathway and directly increasing the transcriptional activity of RARβ [47]. Fenretinide inhibited the proliferation of ovarian cancer cells in vitro and RARβ expression appeared to be associated with this effect [9]. Additionally, apoptosis of neuroblastoma cell lines was blocked by two retinoic acid receptor (RAR)-β/γ-specific antagonists, but not by a RARα-specific antagonist [48]. Fenretinide induces apoptosis in the human RPE cell line ARPE-19 and RARs mediate this process by regulating ROS generation as well as the expression of Gadd153 and HO-1 [49]. However, in this case the specific RARα-specific antagonist AGN194301 could block fenretinde-induced apoptosis [49]. While fenretinide affects the levels of SCD and DES1 in cells and in tissues, the mechanism of modulation is unclear [23,29]. Human retina-derived cells in culture show differences in expression of RAR receptors. ARPE-19 cells express RARα, RARβ and RARγ, while Y79 retinoblastoma cells do not express RARβ [48,49]. Clonetics HRPE primary human cells specifically express only RARβ [50]. Therefore, we wanted to elucidate if fenretinide and MPR could activate or antagonize RARβ and to determine their EC_50_ and IC_50_ concentrations using the GeneBLAzer RAR-beta DA assay, a cell based FRET assay that specifically measures the kinetics of RARβ receptor binding. The positive controls (all-*trans*- and 9-*cis*-retinoic acid) performed as expected and demonstrated high affinity binding (0.31 and 0.28 nM respectively). Meanwhile, fenretinide and MPR were much less potent (EC_50_ = 0.5 and 1.0μM, respectively; EPR had the least affinity with EC_50_ = 4.40 μM, Table 1). The antagonist assay data demonstrated that these phenylretinamides (MPR and EPR) as well as the RBP4 specific ligand A1120 cannot compete with the binding natural ligand all-*trans*-retinoic acid at concentrations 0.5–1.5 nM while fenretinide has an IC_50_ = 10 μM (Fig 4). These data are in good agreement with published data on binding of fenretinide and its metabolites to RARs and corroborate the notion that their effects on cell survival are RAR-independent [33].

**Table 1:** EC50 value comparison.

Compound	EC_50_	EC_50_ from Invitrogen
at-RA	0.31 nM	0.58 nM
9c-RA	0.28 nM	0.3 nM
HPR	0.56 μM	0.22 μM
MPR	1.01 μM	
EPR	4.40 μM	

## Conclusions

We evaluated the inhibitory effects of MPR and 3-keto-HPR, major physiological metabolites of fenretinide, on the known fenretinide targets BCO1, SCD1 and DES1 enzymes. Fenretinide is known to have a breadth of effects on cell homeostasis, interfering with retinol binding and transport, cell survival, inducing apoptosis in cancer and RPE cells, and improving insulin sensitivity and glucose homeostasis *in vitro* and *in vivo* [22,23,34,36,49]. Such a pleiotropic variety of effects of fenretinide and its metabolites is achieved by their various molecular targets, and deconvolution of the effects of fenretinide and its metabolites has proven to be quite a challenging endeavor. However, its effects on insulin sensitivity and glucose homeostasis are now mostly attributed to DES1 inhibition and concomitant decrease in ceramide synthesis [23,42]. Fenretinide-induced cell apoptosis is mediated by different mechanisms, with some being RAR-dependent and some not [14,47,49]. Fenretinide disruption of retinol transport in the eye and the concomitant inhibition of formation of cytotoxic bisretinoids are now largely attributed to its effects on RBP4 retinol transport [25,51]. In our experiments, we did not see changes in A2E bisretinoid accumulation in aged *Bco1*^*-/-*^ mice compared to C57BL6 mice, and therefore we don’t expect BCO1 to be a major local source of all-*trans*-retinal under vitamin A-sufficient diet. We found that MPR is a specific inhibitor of BCO1, but not of SCD1 and DES1. As MPR binds to RBP4, but with lower affinity than retinol or fenretinide, it could be used to target BCO1 *in vivo*. We previously demonstrated that fenretinide induces an ubiquitin-dependent proteasomal degradation of SCD1 in ARPE-19 cells. 3-keto-HPR inhibition of SCD1 expression could follow a similar mechanism [29]. Additionally, as seen from our current and previous data [29,33,49], the effect of fenretinide and 3-keto-HPR on SCD expression cannot be explained by RAR-dependent mechanisms. In contrast, the nonretinoid RBP4-specific ligand A1120 does not affect significantly any of the studied enzymes, though it could weakly affect specificity of DES1 enzyme. The compound A1120 is, so far, the best candidate to target RBP4 in isolation *in vivo* and therefore could be more advantageous to reduce circulating retinol levels in humans. On the other hand, 3-keto-HPR exerts a stronger inhibitory effect on all the studied enzymes than the parent compound in our experiments and shows the most potential to efficiently induce cancer cell apoptosis *in vivo* [33,34]. Thus, care should be taken when fenretinide is used *in vivo* as an inhibitor to account for these and other pleiotropic effects.

## Supporting information

S1 Table Mass spectrometry data for Fig 2B. Area under the curve values (AUC) for palmitoleic (column C) and palmitic acid (column D) measured by Micromass Q-Tof micro mass spectrometer are presented for five treatment groups (DMSO, 4-HPR, MPR, 3-keto-HPR, A1120).
(XLSX)

S2 Table Mass spectrometry data for Fig 3. AUC values for C18 ceramide/C18 dihydroceramide (Sheet 1), C16 ceramide/ C16 dihydroceramide (Sheet 2) and protein concentration (Sheet 3) measured by AB Sciex API2000 triple quadrupole mass spectrometer are presented for five treatment groups (DMSO, 4-HPR, MPR, 3-keto-HPR and A1120).
(XLSX)

## Abbreviations

3-keto-HPR: 4-oxo-N-(4-hydroxyphenyl)retinamide
4-HPR: N-[4-hydroxyphenyl]retinamide
9c-RA: 9-*cis*-retinoic acid
A2E: N-retinylidene-N-retinylethanolamine
at-RA: all-*trans*-retinoic acid
BCO1: beta-carotene oxygenase 1
DES1: dihydroceramide Δ4-desaturase 1
EPR: N-[4-ethoxyphenyl]retinamide
MPR: N-[4-methoxyphenyl]retinamide
RBP4: plasma retinol-binding protein
SCD1: stearoyl-CoA desaturase 1
TTR: transthyretin
